# Role of *Bacillus subtilis* exopolymeric genes in modulating rhizosphere microbiome assembly

**DOI:** 10.1186/s40793-024-00567-4

**Published:** 2024-05-14

**Authors:** Caroline Sayuri Nishisaka, João Paulo Ventura, Harsh P. Bais, Rodrigo Mendes

**Affiliations:** 1 Embrapa Environment, Jaguariúna, SP Brazil; 2 Graduate Program in Agricultural Microbiology, College of Agriculture “Luiz de Queiroz”, University of São Paulo, Piracicaba, SP Brazil; 3 Department of Plant and Soil Sciences, University of Delaware, Newark, DE USA; 4 Ammon Pinizzotto Biopharmaceutical Innovation Center (BPI), Newark, DE USA

**Keywords:** PGPR, *Bacillus subtilis*, *TasA*, *EPS*, Dilution to extinction, Rhizosphere

## Abstract

**Background:**

*Bacillus subtilis* is well known for promoting plant growth and reducing abiotic and biotic stresses. Mutant gene-defective models can be created to understand important traits associated with rhizosphere fitness. This study aimed to analyze the role of exopolymeric genes in modulating tomato rhizosphere microbiome assembly under a gradient of soil microbiome diversities using the *B. subtilis* wild-type strain UD1022 and its corresponding mutant strain UD1022^eps−TasA^, which is defective in exopolysaccharide (*EPS*) and *TasA* protein production.

**Results:**

qPCR revealed that the *B. subtilis* UD1022^eps−TasA−^ strain has a diminished capacity to colonize tomato roots in soils with diluted microbial diversity. The analysis of bacterial β-diversity revealed significant differences in bacterial and fungal community structures following inoculation with either the wild-type or mutant *B. subtilis* strains. The *Verrucomicrobiota*, *Patescibacteria,* and *Nitrospirota* phyla were more enriched with the wild-type strain inoculation than with the mutant inoculation. Co-occurrence analysis revealed that when the mutant was inoculated in tomato, the rhizosphere microbial community exhibited a lower level of modularity, fewer nodes, and fewer communities compared to communities inoculated with wild-type *B. subtilis*.

**Conclusion:**

This study advances our understanding of the EPS and TasA genes, which are not only important for root colonization but also play a significant role in shaping rhizosphere microbiome assembly. Future research should concentrate on specific microbiome genetic traits and their implications for rhizosphere colonization, coupled with rhizosphere microbiome modulation. These efforts will be crucial for optimizing PGPR-based approaches in agriculture.

**Supplementary Information:**

The online version contains supplementary material available at 10.1186/s40793-024-00567-4.

## Background

The rhizosphere microbiome is intricately linked with the host plant [1–3] and is primarily modulated according to the host genotype [4–6] and, consequently, by the plant exudate profile [7–9]. In turn, the rhizosphere microbial community provides readily available nutrients for plant absorption, along with other molecules such as phytohormones and secondary metabolites, which enhance host development and health [10, 11].

The symbiotic relationships between the rhizosphere microbiome and plants can also lead to intricate connections within microbial communities, ultimately benefiting the host plant [12]. For instance, the *Bacillus subtilis* strain UD1022 can colonize *Arabidopsis thaliana* roots, establishing mutualistic interactions [13]. As the host plant secretes fixed carbon through root exudates to nourish the bacteria, *B. subtilis* in turn facilitates rhizobacterium colonization, providing the plant with growth-promoting traits [13]. *B. subtilis* is one of the most studied gram-positive plant growth-promoting rhizobacteria (PGPR) [14, 15], and it has great agricultural and ecological importance [16–19]. Their ability to induce plant development and protect against pathogens and abiotic stresses has been widely explored [20–25]. *B. subtilis* promoted plant growth in tomato [25], cucumber [26], and wheat [27] and conferred resistance against the soil-borne pathogen *Rhizoctonia solani* in cotton [28] and *Pseudomonas syringae* pv. *tomato* in *Arabidopsis* [29]. Martins et al. [20] and Allard-Massicotte et al. [13] showed that *B. subtilis* and *B. amyloliquefaciens* can also induce plant drought tolerance by forming biofilms in bean and *Arabidopsis* roots through bacterial exopolysaccharide (*EPS*) secretion. Most importantly, various *B. subtilis* strains are considered generalists for multiple crops [25–29]. The application of plant growth-promoting rhizobacteria (PGPR) in agricultural settings not only impacts plant performance but also affects the resident soil microbiome. For instance, the use of *bacillus-based* products in crops such as tobacco [30], lettuce [31], and strawberry [32] can increase bacterial diversity in rhizosphere soils.

*EPS* constitute an enclosed matrix produced by microbial multicellular aggregates and serve as the primary component of biofilms [33–35]. Along with EPS, *TasA* is a major proteinaceous component of *B. subtilis* biofilms [36–39]. In addition to affecting motility and chemotaxis, *TasA* plays a role in *B. subtilis* rhizosphere colonization [13, 35, 39], which provides the host plant with an extra barrier against potential soil-borne pathogens and drought tolerance [40–42]. Knocking out the ability of *B. subtilis* to form biofilms by constructing mutant strains is an efficient way to understand its role in plant health and development [35, 43–46]. For instance, *Bacillus* spp. mutated models defective in *YtnP* (lactonase-homolog protein-encoding gene) [44], *EPS*, and *TasA* protein-encoding genes (*tapA– sipW–tasA* operon and *bslA* gene) [45, 46] have been employed in previous studies to elucidate their role in biofilm formation, interactions with the soil microbiome, and their efficacy in antagonizing pathogens.

Studies have reported the effects of mutant *B. subtilis* on plant growth promotion and protection, including sporulation [44], surfactin [45], and flagellar [13, 47] mutants, but the effects of these mutants on rhizosphere microbiome assembly have not yet been properly considered. In this study, *EPS* and *TasA* double mutants of *B. subtilis* were used to investigate the impact of exopolymeric genes on the modulation of rhizosphere microbiome assembly. The use of a mutant strain was combined with the dilution-to-extinction approach to assess the assembly of bacterial and fungal communities in the tomato rhizosphere under a gradient of soil microbial diversity.

## Materials and methods

### Soil microbial diversity dilution

Soil samples were collected from the "UD Fresh to You" farm (39°40′04.2″N 75°45′03.5″W) at the University of Delaware. The specific soil type used was Delanco silt loam, which had previously been cultivated with organic tomatoes and was identified through the Web Soil Survey [48]. The dilution-to-extinction method [49] was employed to obtain soils with the microbial diversity gradient used in the bioassays. Initially, 30 kg of sieved (< 2 mm sieve) and dried soil was divided into three bags, each containing 10 kg of soil. The soil in the bags was autoclaved four times at 120 °C and 1 atm pressure for 60 min. The serial dilution process involved suspending 450 g of natural soil (dry weight) in 900 mL of autoclaved deionized water, resulting in a concentration of 0.5 g mL^−1^ (10^–1^ soil dilution). Subsequently, 100 mL of the 10^–1^ dilution was transferred to 900 mL of autoclaved deionized water to obtain 10^–2^ soil dilutions. This serial dilution process was repeated until a soil dilution of 10^–6^ was reached, following the methods described by Wertz et al. [50] and Souza et al. [51]. Three dilutions were selected for use in the experiment: 10^–1^, 10^–3^, and 10^–6^, in addition to the natural and autoclaved soils. To obtain the microbial diversity gradient across treatments, pots with 200 g of soil received 40 mL of each soil suspension, 10^–1^, 10^–3^, or 10^–6^, and the natural and autoclaved soils received 40 mL of sterilized ultrapure water. Pots were placed in a climatized chamber set at 25 °C, with a photoperiod of 12 h light and 12 h dark. The pots were incubated under these conditions for six weeks, allowing the establishment of the microbiome before the experiment [52].

### *Bacillus subtilis* strains and inoculum preparation

The *B. subtilis* strain UD1022^eps−TasA−^, which is defective for the *EPS* and *TasA* genes, was obtained in a previous study [53]. Wild-type *B. subtilis* was cultivated on Luria broth (LB) agar plates, and its respective mutant was cultivated on LB supplemented with 5 µg mL^−1^ tetracycline and 1 µg mL^−1^ erythromycin. The streaked plates were then incubated at 37 °C for 24 h. Subsequently, individual bacterial colonies were transferred to LB liquid media supplemented with antibiotics, as was the case for the mutant strain UD1022^eps−TasA−^, and incubated in a shaker at 150 rpm for 6 h at 37 °C. After the incubation period, the *B. subtilis* cultures were washed and resuspended in autoclaved distilled water. Bacterial cultures were grown until they reached a concentration of 10^8^ cells mL^−1^. Seeds were disinfected by immersion in a 3% sodium hypochlorite solution and shaken for 30 s, followed by thorough rinsing with ultrapure water. Subsequently, the seeds were briefly soaked in 70% ethanol and shaken for 1 min, followed by another extensive rinse with ultrapure water. After disinfection, 1 g of tomato seeds was mixed with the bacterial suspension (10^8^ cells g^−1^) and kept for 1 h in a shaker at 150 rpm before planting. A boost dose of 1 mL (10^8^ cells mL^−1^) per plant was used on the 16th day of the experiment. In the control treatment, the seeds or plants were treated with autoclaved distilled water.

### Tomato bioassay and experimental design

**Fig. 1:**
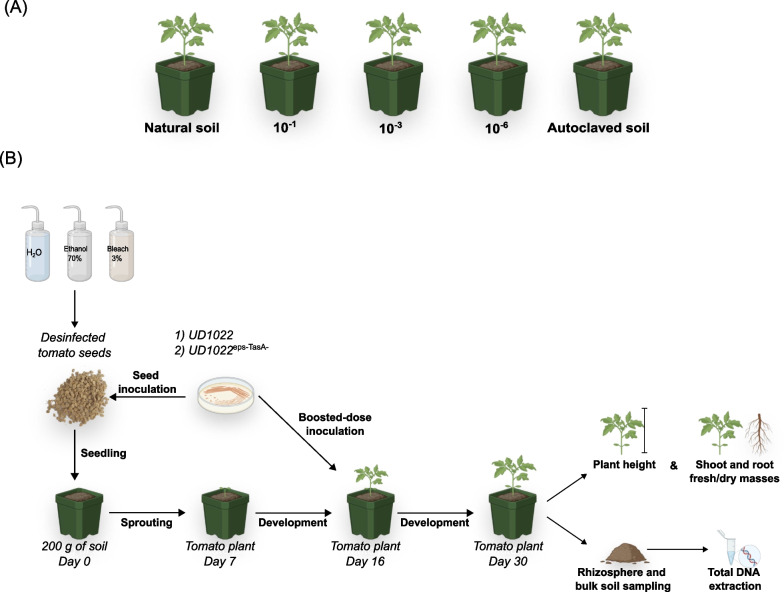
Plant bioassay experimental design and timeline. **A** Each treatment, including plants inoculated with UD1022, plants inoculated with UD1022^eps−TasA−^ and non-inoculated plants (control), was cultivated in soils with five different levels of microbial diversity: natural soil, 10^–1^ dilution, 10^–3^ dilution, 10^–6^ dilution, and autoclaved soil. Pots without plants (bulk soil) were also used as a control. **B** Bioassay timeline showing bacterial inoculation, sampling, and analyses

The plant bioassay used the tomato cultivar “Amish Paste” with four different treatments: (i) plants inoculated with UD1022, (ii) plants inoculated with UD1022^eps−TasA−^, (iii) non-inoculated plants (control), and (iv) pots without plants (bulk soil) (Fig. 1A). Each treatment was performed using five different levels of soil microbial diversity: natural soil, 10^–1^ dilution, 10^–3^ dilution, 10^–6^ dilution, and autoclaved soil. Thus, considering four treatments, soils with five levels of microbial diversity, and five replicates, 100 pots were used in the experiment. Each pot (8 × 6.7 cm) contained 200 g of soil (dry mass), and the plants received at least 10 tomato seeds, which were thinned after five days to leave just one plant per pot. The experiment was conducted using a randomized complete block design. Thirty days after germination, the entire root system was harvested by carefully removing the plants from the pots and gently shaking them to remove excess soil from the root system. The root-adhered soil (i.e., rhizospheric soil) was collected, transferred to 1.5 mL microtubes, and stored at -20 °C before downstream analyses. Various plant growth parameters, including plant height, root fresh and dry masses, and shoot fresh and dry masses, were measured and collected for further data analyses (Fig. 1B).

### Soil microbiome genomic DNA extraction and metataxonomic analysis

Rhizosphere and bulk soil samples were subjected to DNA extraction using the DNeasy PowerSoil® Kit (QIAGEN) according to the manufacturer’s instructions. The quality and concentration of the extracted DNA samples were evaluated using a NanoDrop spectrophotometer. In addition, to verify the integrity of the DNA, electrophoresis was performed on a 1.5% agarose gel at 80 V/400 mA for 45 min. To ensure sample DNA concentrations, quantification was performed using a QUBIT® fluorometer.

DNA samples from all five replicates per treatment (a total of 100 samples) were subjected to amplicon sequencing using the Illumina MiSeq platform at the Delaware Biotechnology Institute in Newark, Delaware, USA. Sequencing of the V4 region of the bacterial 16S rRNA gene was performed using the following primer pairs: 515F (5′-GTGYCAGCMGCCGCGGTAA-3′) [54] and 806R (5′- GGACTACNVGGGTWTCTAAT-3′) [55], and sequencing of the ITS1 region of the fungal ITS gene was performed using the following primer pairs: ITS1f (5′-CTTGGTCATTTAGAGGAAGTAA-3′) and ITS2 (5′-GCTGCGTTCTTCATCGATGC-3′) [56].

### Rhizosphere and bulk soil *Bacillus* quantification using quantitative polymerase chain reaction (qPCR)

Initially, *B. subtilis* UD1022 genomic DNA was used to prepare a qPCR standard curve. Total DNA was extracted from *B. subtilis* strain UD1022 liquid cultures using the DNeasy® UltraClean® Microbial Kit (QIAGEN) according to the manufacturer’s instructions. The quality of the total extracted DNA was assessed using a NanoDrop® ND-2000 Spectrophotometer (Thermo Fisher Scientific, Wilmington, DE, USA). DNA was quantified using a QUBIT® 2.0 fluorometry system (Thermo Fisher Scientific, Wilmington, DE, USA). DNA was stored at -20 °C for subsequent analyses.

To prepare the standard curve, serial dilutions of *B. subtilis* UD1022 genomic DNA were prepared at a 1:10 ratio. The starting concentration of genomic DNA used for the dilutions was standardized at 10 ng μL^−1^. The target DNA was amplified using primers designed for the *gyrB* gene, which encodes DNA gyrase subunit B. The primers gyrB_5234_F (5′-CGGTCGTAAACGCACTATC-3′) and gyrB-5391_R (5′-AGGGTCCGGGACAAAATGTGTCG-3′) were adapted from Xie et al. [57]. Each qPCR reaction had a final volume of 10 μL and included the following components: 5 μL of PerfeCTa SYBR® Green SuperMix (Quantabio, Baverly, MA, USA), 0.2 μL of each primer (10 mM), 1 μL of template DNA, and 3.6 μL of ultrapure water. The reaction conditions were adapted from Xie et al. [54] and involved an initial denaturation step at 95 °C for 10 min, followed by 40 cycles of denaturation at 95 °C for 30 s, annealing at 61 °C for 35 s, and extension at 72 °C for 40 s. Melting curve collection was performed at the end of the cycling program. Distilled water was used as the non-template control. The qPCR assay was performed in triplicate for each dilution to ensure the accuracy and reproducibility of the results. The standard curve served as a reference to quantify the abundance of *Bacillus* in soil samples by interpolating their Ct values onto the curve and then converting to the number of *gyrB* per gram of soil. For *Bacillus subtilis* quantification in the rhizosphere and bulk soil samples, qPCR was performed with standard curves under the same conditions described above.

### Data processing and statistical analyses

Bioassay data, including plant height, shoot and root dry mass, and qPCR (number of copies of the gyrB gene), were compared using the *Scott–Knott* test (P < 0.05). To generate amplicon sequence variants (ASVs) from both genes, 16S rRNA and ITS, the raw data were processed using Dada2 version 1.21.0 [58]. The primers were removed using Cutadapt version 3.4. [59] Quality control was performed and reads with low quality (Q20 or lower) were discarded, followed by taxonomic assignment using the Silva (v. 138.1) [60, 61] and UNITE (v. 9.0) databases [62–64]. To assess α-diversity, the Chao1 and Shannon indices were calculated. β-diversity was assessed using the Bray‒Curtis distance. Principal coordinate analysis (PCoA) was employed to visualize the similarity matrix among various soil diversity dilutions and treatments. In both analyses, rarefied and normalized data were used. The significance and effect size β-diversity were determined using the *vegan* package (v. 2.6-4) through permutation-based analysis (MANOVA) with the "adonis()" function [65]. To identify differentially abundant taxa among the treatment groups, ANOVA-like differential expression analysis (ALDEx2) [66] was performed using the "run_aldex()" function from the *microbiomeMarker* package (v. 1.28.1) [67]. In addition, a co-occurrence network analysis was performed using the PhyloSmith package (v. 1.0.6) [68] based on Spearman's pairwise correlation. To mitigate the influence of rare ASVs, ASVs occurring fewer than 20 times in each treatment with a relative abundance greater than 30% were excluded. Significant interactions were identified using Spearman pairwise correlations, with p-values less than 0.001 indicating robust associations. The nature of the co-occurrence relationships, whether negative or positive, was determined based on the strength of the correlation. In the network, each ASV represents a node, and the edges represent the correlations among the ASVs. The network layout was generated using the Fruchterman-Reingold algorithm in Gephi software (v. 0.10) [69].

## Results

### Quantification* of Bacillus subtilis* in the tomato rhizosphere

**Fig. 2:**
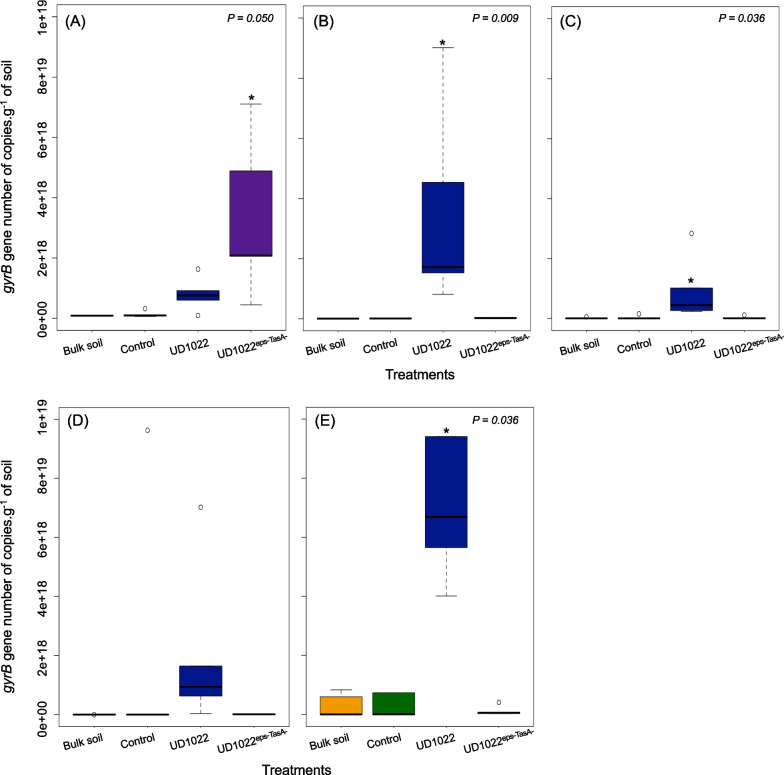
Boxplot of the *gyrB* gene (DNA gyrase subunit B coding gene) and qPCR quantification of *B. subtilis*. **A** Natural soil. **B** Soil dilution 10^–1^. **C** Soil dilution 10^–3^. **D** Soil dilution 10^–6^. **E** Autoclaved soil. Bulk soil = non-inoculated soil; Control = non-inoculated plants; UD1022 = plants inoculated with wild-type *B. subtilis*; and UD1022^eps−TasA−^ = plants inoculated with mutant *B. subtilis*. The Scott–Knott test for pairwise comparisons of means was performed considering a 95% familywise confidence level (P < 0.05). * Indicates significant differences between treatments

Quantitative real-time PCR (qPCR) was performed to quantify the abundance of *B. subtilis gyrB* gene copies in the rhizosphere and bulk soils. In natural soil, a greater amount of *B. subtilis gyrB* was detected in the treatment group inoculated with the UD1022^eps−TasA−^ strain (Fig. 2A). At 10^–1^, 10^–3^, and autoclaved soil dilutions, *B. subtilis* UD1022 was more abundant in the wild-type soil than in the other treatments (Fig. 2BCE).

### Inoculation of *Bacillus subtilis* strains and plant performance

**Fig. 3:**
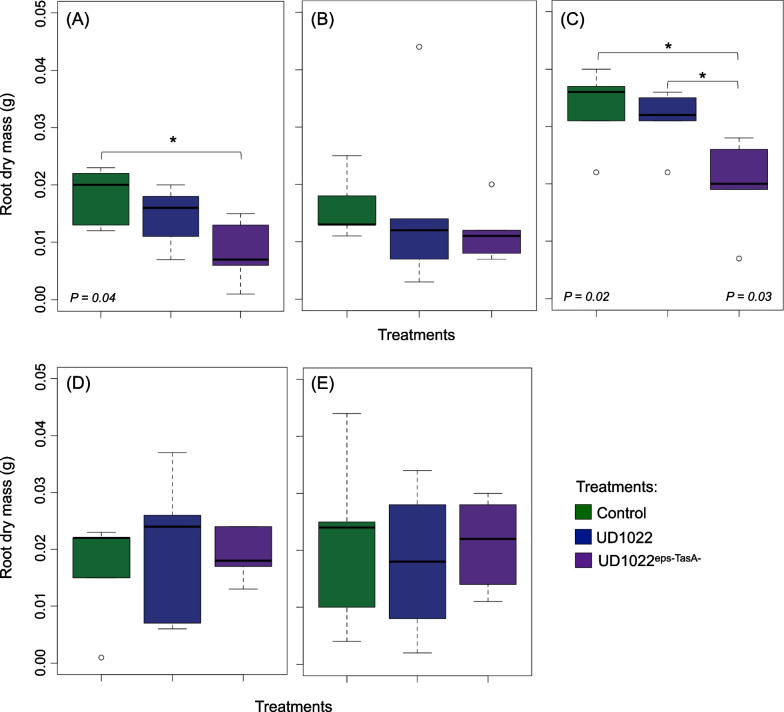
Boxplot of the root dry mass of tomato plants 30 days after germination. **A** Natural soil; **B** Soil dilution 10^–1^; **C** Soil dilution 10^–3^; **D** Soil dilution 10^–6^; and **E** Autoclaved soil. Control = non-inoculated plants; UD1022 = plants inoculated with wild-type *B. subtilis*; and UD1022^eps−TasA−^ = plants inoculated with mutant *B. subtilis*. The Scott–Knott test for pairwise comparisons of means was performed considering a 95% familywise confidence level (P < 0.05). Asterisks (*) indicate significant differences between treatments

Significant differences in plant growth were observed for root dry mass with *Bacillus* inoculation (Fig. 3). According to pairwise comparisons, compared with non-inoculated plants (controls), tomato plants inoculated with the mutant strain UD1022^eps−TasA−^ showed a reduction in root dry mass when grown in natural soil (Fig. 3A) or at a soil dilution of 10^–3^ (Fig. 3C). At a soil dilution of 10^–3^, plants inoculated with the wild-type strain UD1022 showed significantly increased root growth compared with plants inoculated with the mutant strain (Fig. 3C). No differences were observed across treatments for plant height (Additional file 1: Figure S1A to E) or shoot dry mass (Additional file 1: Figure S1F to G). Within each treatment, plants generally grew better in soils with diluted microbial diversity (Additional file 1: Figure S2).

### Impact of the *Bacillus subtilis* strain UD1022 on rhizosphere microbiome assembly

**Fig. 4:**
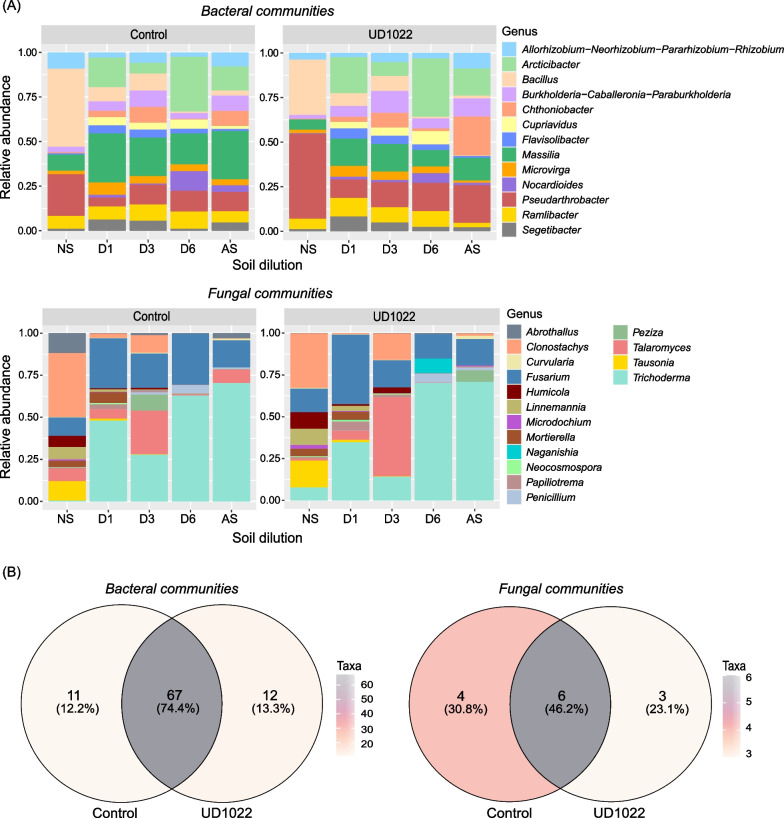
Composition of bacterial and fungal communities in the tomato rhizosphere. **A** Relative abundance of bacteria and fungal genera across soils with a microbial diversity gradient. NS = natural soil, D1 = soil dilution 10^–1^, D3 = soil dilution 10^–3^, D6 = soil dilution 10^–6^, and AS = autoclaved soil. **B** Venn diagram of bacterial and fungal taxa. Control = non-inoculated plant; UD1022 = plants inoculated with wild-type *B. subtilis*

The bacterial community in natural soils was dominated by *Bacillus* and *Pseudarthrobacter* in non-inoculated or inoculated soils, respectively, with strain UD1022 (Fig. 4A). These two bacterial taxa decreased in relative abundance in soils with diluted microbial diversity (Fig. 4A). *Bacillus* inoculation changed the relative abundance of specific bacterial and fungal groups in the tomato rhizosphere. For example, inoculation with strain UD1022 increased the relative abundance of *Pseudarthrobacter* in natural soil and *Chthoniobacter* in autoclaved soil (Fig. 4A). Twelve bacterial taxa were exclusively found in the rhizosphere of plants inoculated with the UD1022 strain (Fig. 4B), including *Mucilaginibacter* spp. ASV-0058, *Curtobacterium* spp. ASV-0097, *Kaistia* spp. ASV-0098, *Sumerlaea* spp. ASV-0135, *Nocardioides* spp. ASV-0181, and *Methylorosula* spp. ASV-0216 (Fig. 4B).

The fungal community was dominated by *Clonostachys* in natural soils inoculated with or without strain UD1022 (Fig. 4A). In soils with diluted microbial diversity, the communities were dominated by *Trichoderma* and *Fusarium* (Fig. 4A). Moreover, inoculation with the UD1022 strain significantly increased the relative abundance of *Talaromyces* at a dilution of 10^–3^ (Fig. 4A). Compared with that in the control treatment, the abundance of *Talaromyces* increased at a dilution of 10^–3^ when UD1022 was inoculated (Fig. 4A). *Trichoderma ghanense* ASV-0001, *Trichoderma* spp. ASV-0003, and *Cryptococcus laurentii* ASV-0025 were exclusively detected in the rhizospheres of plants inoculated with strain UD1022 (Fig. 4B).

The α-diversity analysis using the Shannon index and HSD test (P < 0.05) revealed significant differences in the bacterial community across all the soil dilutions, except between the 10^–6^ soil dilution and autoclaved soil (Additional file 1: Figure S3A). As expected, the natural soil exhibited the highest bacterial diversity, followed by the 10^–1^, 10^–3^, 10^–6^ dilutions, and autoclaved soil. The same pattern was observed in the fungal community (Additional file 1: Figure S3B).

Soil microbial diversity dilution significantly affected the assembly of the rhizosphere microbiome in plants inoculated with or without strain UD1022. The relative abundance of the *Bacillota* phylum (Additional file 1: Figure S4), particularly the *Bacillus* genus (Additional file 1: Figure S4), decreased as the soil diversity decreased. The abundances of the phyla *Acidobacteria* and *Crenarchaeota* exhibited the same pattern; they decreased with soil microbial dilution and were not detected in the most diluted or autoclaved soil (Additional file 1: Figure S4). Conversely, the relative abundances of the phyla *Bacteroidota*, *Planctomycetota*, and *Pseudomonadota* increased with decreasing soil microbial diversity (Additional file 1: Figure S4).

For the fungal community, the relative abundance of the phyla *Mortierellomycota* and *Basidiomycota* decreased as the soil diversity decreased (Additional file 1: Figure S5). In the control treatment and in plants inoculated with the mutant UD1022^eps−TasA−^, they were not detected in soil diluted 10^–6^ or autoclaved soil (Additional file 1: Figure S5). The phylum *Chytridiomycota* was detected only in the 10^–1^ dilution soil, and *Rozellomycota* was exclusively found in plants growing in natural soil inoculated or not inoculated with *Bacillus* strains (Additional file 1: Figure S5).

**Fig. 5:**
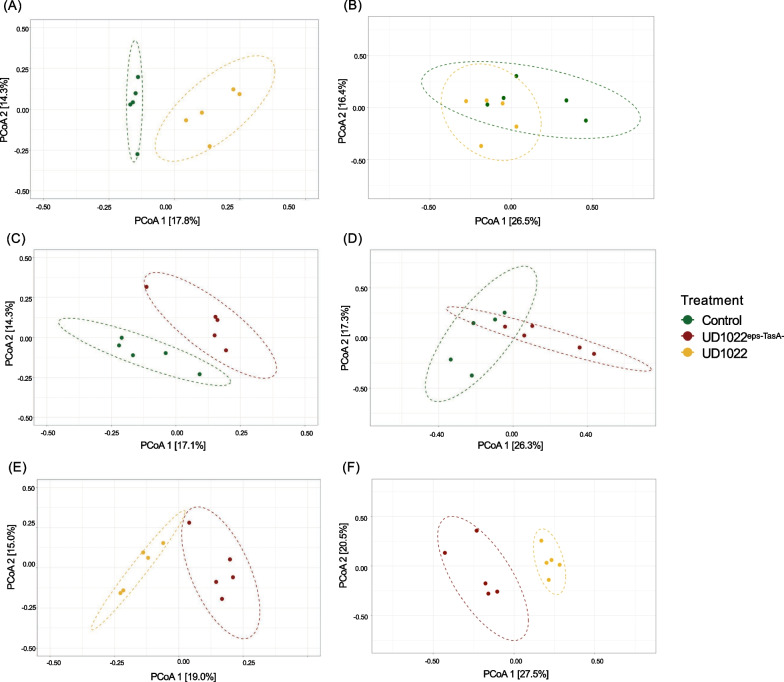
PCoA analysis of the rhizosphere microbiome of tomato plants cultivated in autoclaved soil. Statistical pairwise comparisons were performed using the Adonis method (P < 0.05, permutation = 999). **A** Comparison of bacterial communities between the control group and the UD1022 treatment group (P = 0.008). **B** Comparison of fungal communities in the control group versus the UD1022 treatment group (P = 0.025). **C** Comparison of the bacterial communities of the control group and the UD1022^eps−TasA−^ treatment group (P = 0.011). **D** Comparison of fungal communities in the control group versus the UD1022^eps−TasA−^ treatment group (P = 0.012). **E** Comparison of the bacterial communities of the UD1022^eps−TasA−^ and UD1022-treated groups (P = 0.009). **F** Comparison of fungal communities in the UD1022^eps−TasA−^ versus UD1022 treatment groups (P = 0.011). Control = non-inoculated plants; UD1022 = plants inoculated with the wild-type strain of *B. subtilis;* and UD1022^eps−TasA−^ = plants inoculated with the mutant strain of *B. subtilis*

To better understand how inoculation influenced the assembly of bacterial and fungal communities in the plant rhizosphere under low microbial diversity, β-diversity measurements were separately conducted using samples from each dilution (Additional file 1: Figures S6 and S7). Significant changes in the composition of the bacterial and fungal communities were observed when *Bacillus* strains were inoculated into autoclaved soil (Fig. 5A, B). The control and UD1022 treatments in natural soil and at dilutions of 10^–1^, 10^–3^, and 10^–6^ did not significantly change the bacterial or fungal β-diversity (Figures S6 and S7).

### Role of *EPS* and *TasA* in rhizosphere microbiome assembly

Inoculation with the mutant strain UD1022^eps−TasA^ decreased the abundance of the genus *Rhizobium* in the rhizosphere microbiome of natural soil compared with that in non-inoculated plants (Additional file 1: Figure S8A). Inoculation with the mutant strain resulted in a reduced abundance of *Abrothallus* and *Clonostachys* in the natural soil (Additional file 1: Figure S8B). Several bacterial and fungal taxa were detected only when the wild-type strain UD1022 was inoculated (Additional file 1: Figure S9), including *Pedobacter* spp. ASV-0024 and ASV-0126, *Gemmatimonas* spp. ASV-0066, *Planococcaceae* spp. ASV-0078, *Flavisolibacter* spp. ASV-0089, *Curtobacterium* spp. ASV-0097, *Mesorhizobium* spp. ASV-0110, *Nocardioides* spp. ASV-0181, and *Methylorosula* spp. ASV-0216. However, some microbial taxa were exclusively detected when the mutant strain UD1022^eps−TasA−^ was inoculated, including *Streptomyces* spp. ASV-0076, *Chthoniobacter* spp. ASV-0079, *Frankia* spp. ASV-0083, *Bryobacter* spp. ASV-0100, *Adhanibacter* spp. ASV-0129, *Parasegetibacter* spp. ASV-0142, *Methylorosula* spp. ASV-0196, *Flavisolibacter* spp. ASV-0222, and *Micropepsaceae* ASV-0091 (Additional file 1: Figure S9).

Analysis of bacterial β-diversity revealed significant differences in the structures of bacterial and fungal communities due to *Bacillus* inoculation. These differences in β-diversity were observed not only in the comparison between non-inoculated and inoculated plants but also between plants inoculated with the wild-type strain UD1022 or the mutant UD1022^eps−TasA−^ (Fig. 5 and Figures S10-S13). These differences were more remarkable in soils with lower microbial diversity (i.e., autoclaved soil) (Fig. 5).

**Fig. 6:**
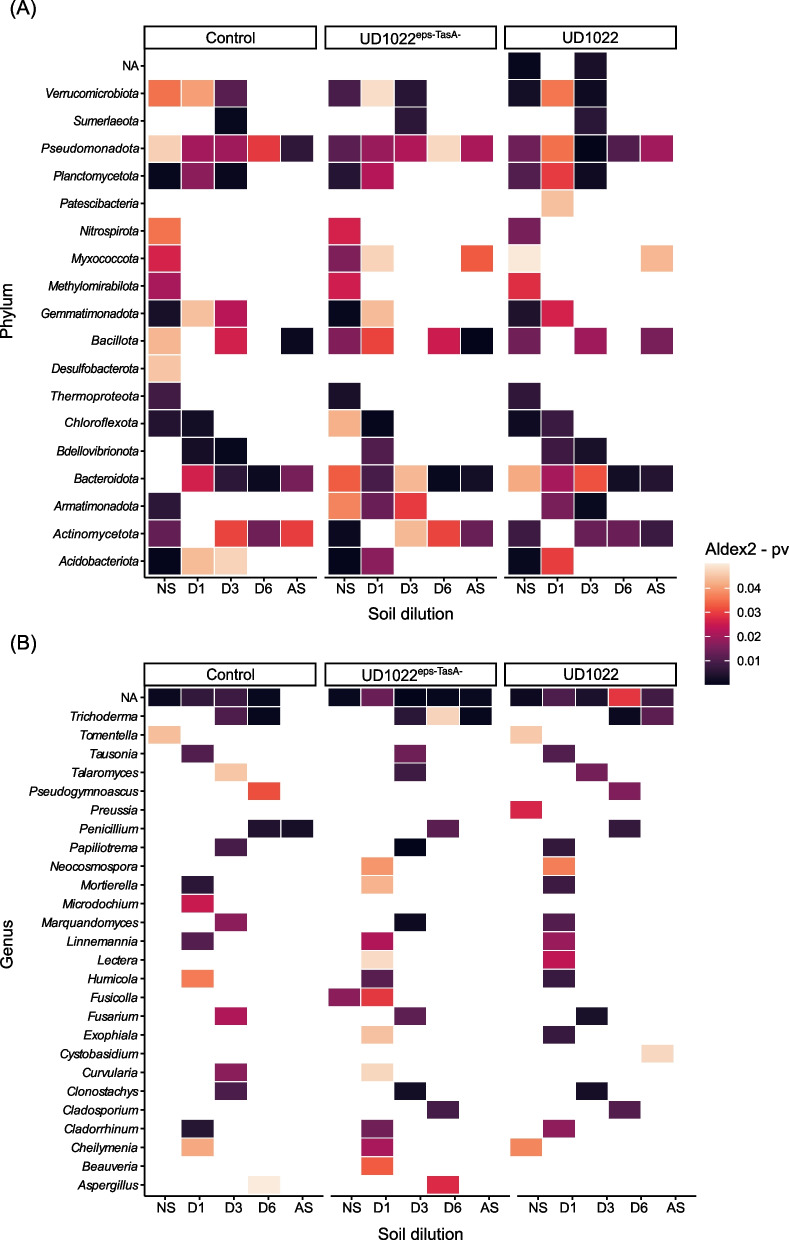
Heatmaps showing variations in the relative abundances of bacteria (**A**) and fungi (**B**) across treatments. ALDEx2 analysis was performed using Monte Carlo distances from the Dirichlet distribution with a *P* cutoff of 0.05. The color spectrum on the heatmap, ranging from dark purple to orange, represents statistically significant disparities in relative abundance (p < 0.05 to 0.01). A value of 0.01 indicates a more pronounced level of differential enrichment. NS = natural soil, D1 = soil dilution 10^–1^, D3 = soil dilution 10^–3^, D6 = soil dilution 10^–6^, and AS = autoclaved soil. Control = non-inoculated plants; UD1022 = plants inoculated with wild-type *B. subtilis*; and UD1022^eps−TasA−^ = plants inoculated with mutant *B. subtilis*

Differential abundance analysis using ALDEx2 was employed to identify microbial taxa that were differentially enriched in plants inoculated with the wild-type strain or the defective mutant lacking *EPS* and *TasA*. Compared with those of the mutants, the abundances of the phyla *Verrucomicrobiota* (P < 0.01), *Patescibacteria* (P < 0.05), *Nitrospirota* (P < 0.02), *Bdellovibrinota* (P < 0.01), *Armatimonadota* (P < 0.01), and *Actinomycetota* (P < 0.01) in the wild-type *B. subtilis* strains were greater than those in the wild-type strain UD1022 (Fig. 6A). Compared with inoculation with the mutant strain, inoculation with the wild-type strain also increased the abundances of specific members of the fungal community, including *Tomentella* (P < 0.05), *Pseudogymnoascus* (P < 0.02), *Preussia* (P < 0.03), *Motirella* (P < 0.01), *Lectera* (P < 0.02), *Humicola* (P < 0.01), *Fusarium* (P < 0.01), *Exophiala* (P < 0.01), and *Cystobasidium* (P < 0.05) (Fig. 6B).

### Co-occurrence network analysis of the rhizosphere microbiome

**Fig. 7:**
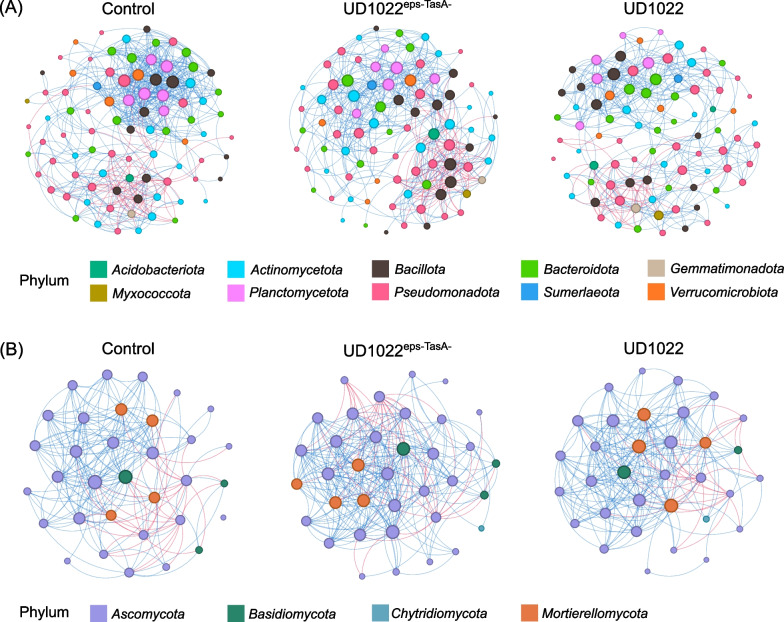
Co-occurrence network of ASVs according to the Fruchterman Reingold distribution. ASVs were filtered considering occurrence > 20 times and > 30% abundance. **A** Bacterial community networks. **B** Fungal community networks. Non-inoculated plant = Control; plant inoculated with wild-type *B. subtilis* = UD1022; and plant inoculated with mutant *B. subtilis* = UD1022^eps−TasA−^

The construction of the bacterial networks revealed that inoculation with *Bacillus* strains affected the complexity of the network (Fig. 7 and Additional file 1: Table S1). The number of edges in the network of plants inoculated with the wild-type strain UD1022 and the mutant UD1022^eps−TasA−^ was reduced compared with that in the network of non-inoculated plants. A decrease in the number of nodes was also observed in inoculated plants, especially when the mutant UD1022^eps−TasA−^ was inoculated (Fig. 7A). Moreover, a greater number of nodes, modularity, and number of communities were observed when UD1022 was inoculated than when UD1022^eps−TasA^ was inoculated (Fig. 7 and Additional file 1: Table S1). In contrast, compared with wild-type inoculation, mutant inoculation resulted in a greater total number of edges, including negative and positive edges (Additional file 1: Table S1). Thus, compared with the control, UD1022^eps−TasA^ inoculation led to a decrease in the number of positive connections and an increase in the number of negative edges, whereas wild-type UD1022 inoculation resulted in a decrease in the number of positive and negative edges (Fig. 7 and Additional file 1: Table S1).

In terms of the fungal community, plants in the control treatment exhibited greater modularity than did those in the rhizosphere of plants inoculated with the wild-type strain UD1022 or the mutant strain UD1022^eps−TasA−^ (Additional file 1: Table S1). Notably, in contrast to the bacterial networks, inoculation with the mutant strain UD1022^eps−TasA−^ improved fungal connections (Fig. 7B). This was evident by an increase in the number of nodes, edges, and average clustering coefficient parameters, surpassing those observed in the control and UD1022 treatments (Additional file 1: Table S1). In addition, compared with the control treatment, UD1022^eps−TasA^ inoculation increased the number of positive and negative connections, whereas UD1022 increased the number of positive edges and decreased the number of negative edges (Additional file 1: Table S1).

## Discussion

Previous studies have shown that the *Bacillus subtilis* strain UD1022 exerts beneficial effects on plant growth and offers protection against plant pathogens [13, 23, 45, 46, 53]. Most experiments involving plant growth-promoting rhizobacteria (PGPR) are conducted using artificial soil or under controlled laboratory conditions [70]. Under such conditions, numerous bacterial isolates exhibit promising traits for plant growth promotion, including siderophore production, phosphate solubilization, and phytohormone synthesis [71]. However, when these microorganisms are applied in more complex systems, such as agricultural soil or under on-farm conditions, many of these traits may be subdued or even remain unexpressed owing to factors such as niche competition, nutrient limitation, antagonistic interactions, and environmental conditions [1, 72, 73].

Therefore, the inoculation of PGPR can encounter various challenges when interacting with the resident soil microbiome, primarily because of the diverse microbial community and the complexity of the relationships present around the roots [73–75]. As plants shape the rhizosphere microbiome according to their needs at each life stage [76, 77], soil diversity is considered an important microbial reservoir for plant root recruitment through exudation [3, 78].

Inoculation with the wild-type strain UD1022 increased ~ 100 to 200-fold the number of *B. subtilis gyrB* genes detected in the rhizosphere of plants growing in soils with diluted microbial diversity compared with that in non-inoculated plants or those inoculated with the mutant strain UD1022^eps−TasA^. This observation suggests that root colonization by *Bacillus* is enhanced in soils with lower microbial diversity because less diverse soils can generate more open environments and less nutrient and niche competition, favoring the establishment of inoculants [79–83]. The inverse relationship between soil microbial diversity and the survival of an invading bacterial pathogen was previously demonstrated using the dilution-to-extinction approach [79].

As expected, in soils with lower microbial diversity, the wild-type strain UD1022 was more efficient at colonizing the rhizosphere than the mutant strain UD1022 ^eps−TasA−^. This observation confirms the importance of the *EPS* and *TasA* genes in root establishment. Surprisingly, in natural soil, a higher number of *gyrB* genes were detected when plants were inoculated with the mutant strain. This difference may be attributed to the naturally higher abundance of *Bacillus* sp. in natural soil, suggesting that inoculation with mutated *B. subtilis* UD1022^eps−TasA^ stimulated the enrichment of native *Bacillus* sp. in the rhizosphere. The effects of *B. subtilis* mutation on biofilm production in soil are not well understood. Moreover, Zhu et al. [84] recently demonstrated a growth-survival fitness trade-off in *B. subtilis* mutants lacking the master regulator sporulation gene (*Spo0A*-) in vitro. Their findings showed that *Spo0A*-null strains exhibited increased growth capacity, both in terms of rate and yield, compared with wild-type *B. subtilis* because of resource reallocation [84]. However, considering that the primer pair employed to detect *B. subtilis* was not strain specific, we were not able to discriminate between soil-resident *B. subtilis* and the inoculated strain UD1022.

Inoculation with the mutant strain UD1022^eps−TasA−^ at a soil dilution of 10^–3^ resulted in plants with reduced root dry mass compared with that of plants inoculated with the wild-type strain. This result implies that the inability of strain UD1022^eps−TasA−^ to form biofilms prevents effective colonization of the plant rhizosphere, resulting in less effective plant growth promotion compared with that of the UD1022 wild-type. The genetic traits of the inoculant, such as the presence of chemoreceptors and mobility in soil, are crucial for establishing the inoculant in the plant rhizosphere [13, 85, 86]. However, in this study, the resident microbial diversity found in natural soil may have played an important role in promoting plant growth [85].

In this context, the dilution-to-extinction method has been applied to better understand the impacts of microbial invasion on the rhizosphere microbiome [49–52, 87]. Such research has shed light on changes in soil microbial communities under biotic disturbances [87]. For example, Ferrarezi et al. [88] demonstrated that the PGPR *Azospirillum brasilense* had a more pronounced beneficial impact on plants growing in soils with lower microbial diversity, as determined by applying the dilution-to-extinction method. It is important to emphasize that the autoclaved soil did not receive any microbial inoculum, and the microbial communities found in this treatment originated naturally from the soil. After autoclaving, the soil was pre-incubated to reach a microbial biomass similar to that of the other treatments but with reduced species richness [52]. Natural ecosystems show variable resistance to invasion by alien species, and this resistance can be related to species diversity in the system [78]. Mawarda et al. [89] reported that the response of the soil microbial community to an introduced organism is also contingent on the nature and extent of the invasion disturbance. This is closely linked to the ecological strategies and functional traits of each invader.

Concurrently, beneficial bacteria and fungi were also enriched when the wild-type strain UD1022 was inoculated. For example, the bacterial genera *Chthoniobacter* and *Pseudarthrobacter* are known to contain endophytic/PGPR strains and species that play a role in the transformation of organic carbon compounds in soil [90–93]. In general, *Pseudogymnoascus, Preussia, Humicola, Fusarium, Exophiala, and Cystobasidium* were the most enriched fungal genera when UD1022 was inoculated. Interestingly, some of these groups, including *Preussia* [94, 95], *Humicola* [96, 97], and *Exophiala* [98, 99], are known growth promoters in rice. On the other hand, the relative abundance of *Actinomycetota*, which is a phylum harboring well-known beneficial bacteria [99–102], decreased in plants inoculated with UD1022^eps−TasA−^ compared to that in plants inoculated with UD1022 (Additional file 1: Figure S4). This observation may imply the importance of the *EPS* and *TasA* genes in synergistic interactions among soil resident microbiome communities. Sun and collaborators [103] demonstrated the importance of *Bacillus* biofilm formation in syntrophic cooperation in soil. Inoculation with the wild-type *B. velezensis* strain SQR9 positively induced plant-beneficial indigenous *Pseudomonas stutzeri* in the cucumber rhizosphere by branched-chain amino acid (BCAA) production compared with a mutant defective in *EPS* and *TasA* [103]. In the present study, the same pattern was observed in soil dilutions 10^–3^ and 10^–6^, where *Pseudomonadota*, a phylum of the *Pseudomonas* genus, was significantly enriched when the UD1022 strain was inoculated compared with the UD1022^eps−TasA−^ strain. The impact of UD1022 inoculation on the bacterial community exhibited a stronger effect on the β-diversity of autoclaved soil than on that of natural soil. This observation suggested that the absence of competition and reduced niche occupancy in autoclaved soil may have allowed the inoculant to significantly alter the composition of the bacterial communities [104]. Mallon et al. [81] demonstrated a similar pattern for foreign microbial invaders when the soil microbial composition was compromised, fitting the paradigm of diversity-invasion effects [80–82, 105, 106], where less diverse communities have limited abilities to use available resources, and consequently, their ability to mitigate external microbial invasion decreases.

Previous studies have highlighted the important effect of the *B. subtilis EPS* and *TasA* genes on social interactions in rhizosphere soil using double-mutated bacteria and soil resident communities [34, 45, 107]. These studies emphasized the effect of the extracellular matrix on the bacterial consortium between two bacterial species and its importance in salt stress tolerance. This study extends this understanding by showing how *EPS* and *TasA* gene knockout in *B. subtilis* affects rhizosphere microbiome assembly.

Biofilm formation is essential for successful rhizosphere colonization, with the *TasA* gene playing a crucial role in stabilizing biofilm membrane dynamics and enabling cellular adaptation, mainly in plant interactions [34, 107, 108]. In this context, compared with UD1022 inoculation, co-occurrence network analysis revealed that inoculation with the mutant strain UD1022^eps−TasA−^ decreased the number of nodes and increased the number of negative interactions in the bacterial network. On the other hand, plants inoculated with UD1022 exhibited denser connections within subcommunities than within the entire network, which could be one of the stages of biofilm production, which includes microcolony formation [108]. The fungal community network was also altered by inoculation with the mutant strain UD1022^eps−TasA−^. While the bacterial network decreased the number of nodes in the presence of the mutant strain, the fungal network showed more nodes than did the network in the presence of the wild-type strain UD1022. Therefore, as bacteria engage with eukaryotes, highlighting the significance of social interactions in the coevolution of fungi and bacteria, this dynamic process fosters specific interactions and the potential generation of metabolites that influence network outcomes [109]. Notably, both the core components of the matrix, *EPS* and *TasA*, significantly contribute to establishing robust interactions with other microorganisms [110].

Taken together, the results of this study underscore the critical role of the *EPS* and TasA genes in *B. subtilis* strain UD1022 for effective plant growth promotion and modulation of soil microbial communities. The presence of these genes significantly influenced microbial β-diversity, especially in less diverse soils, demonstrating their importance in shaping the rhizosphere microbiome. The absence of these genes, as observed in plants inoculated with UD1022^eps−TasA−^, altered the bacterial and fungal communities, demonstrating their role in social interactions and community dynamics. In addition, co-occurrence network analysis revealed that the absence of the *EPS* and *TasA* genes impacted the structure and dynamics of the bacterial networks in the rhizosphere. This study emphasizes that understanding genetic traits such as *EPS* and *TasA* is vital for comprehending how PGPRs interact with the rhizosphere microbiome and, consequently, influence plant health and growth. Further research on specific microbiome genetic traits and their implications for rhizosphere colonization will significantly contribute to the optimization of PGPR-based approaches in agriculture.

### Supplementary Information

**Additional file 1:** The Supplementary Information contains additional data and results.

## Data Availability

The raw amplicon sequencing data are available at the NCBI project number PRJNA1054195. The soil sample access activity is registered at SISGen A5EB05F.
