# Supplementary material for: Role of Bacillus subtilis exopolymeric genes in modulating rhizosphere microbiome assembly
Source: Environ Microbiome. 2024 May 14;19:33. doi: 10.1186/s40793-024-00567-4 (PMC11092206; doi:10.1186/s40793-024-00567-4)
Supplement: Supplementary file 1 — Additional file 1: The Supplementary Information contains additional data and results. [file 40793_2024_567_MOESM1_ESM.docx]

SUPPLEMENTARY MATERIAL

**Role of *Bacillus subtilis* exopolymeric genes in modulating rhizosphere microbiome assembly**

Caroline Sayuri Nishisaka^1,2^, João Paulo Ventura^1,2^, Harsh Bais^3,4^, Rodrigo Mendes^2*^

^1^ Embrapa Environment, Brazil

^2^ Graduate Program in Agricultural Microbiology, College of Agriculture “Luiz de Queiroz”, University of São Paulo, Brazil

^3^Department of Plant and Soil Sciences, University of Delaware, Newark, DE, USA

^4^Ammon Pinizzotto Biopharmaceutical Innovation Center- (BPI), 590 Avenue 1743, Newark, DE 19713

******Corresponding author:* [rodrigo.mendes@embrapa.br](mailto:rodrigo.mendes@embrapa.br)

This file contains:

Supplementary Figures: 1 to 13

Supplementary Table: 1

**Figure S1** Plant height and shoot dry mass comparing the treatments within each gradient of microbial diversity. Plants heights in natural soil (**A**), soil dilution 10^-1^ (**B**), soil dilution 10^-3^ (**C**), soil dilution 10^-6^ (**D**), and autoclaved soil (**E**). Plant shoot dry mass in natural soil (**F**), soil dilution 10^-1^ (**G**), soil dilution 10^-3^ (**H**), soil dilution 10^-6^ (**I**), and autoclaved soil (**J**). Control = non-inoculated plants, UD1022 = plants inoculated with wild-type B. subtilis, and UD1022^eps-TasA-^ = plants inoculated with mutant B. subtilis. The Scott-Knott test pair-wise comparison of means was performed considering 95% family-wise confidence level (P<0.05). Asterisks (*) indicate significant differences between treatments.

**Figure S2** Plant shoot and root dry mass comparing the gradient of microbial diversity within each treatment. Plant shoot dry mass in the control treatment (**A**), in plants inoculated with the wild-type UD1022 (**B**), and in plants inoculated with the mutant UD1022^eps-TasA-^ (**C**). Root dry mass in the control treatment (**D**), in plants inoculated with the wild type UD1022 (**E**), and in plants inoculated with the mutant UD1022^eps-TasA-^ (**F**). The Scott-Knott test pair-wise comparison of means was performed considering 95% family-wise confidence level (P<0.05). Asterisks (*) indicate significant differences between treatments.


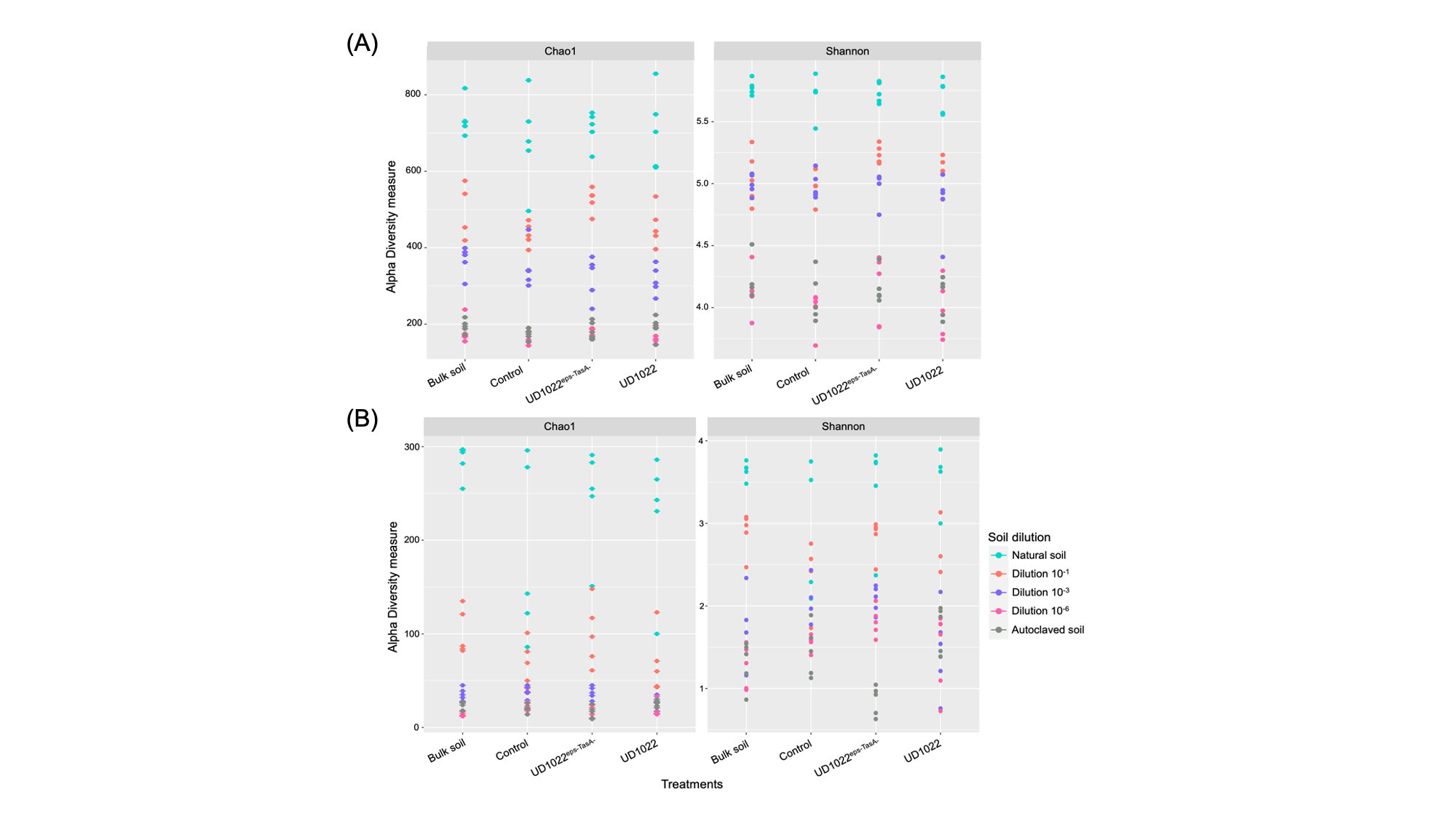


**Figure S3** Chao1 and Shannon diversity. Bacterial community α-diversity based on the V4 region from 16S rRNA gene. According to the HSD test (P<0.05) using Shannon index, all dilutions have statistical diferences in bacterial community, except for the dilution 10^-6^ and autoclaved soil. The highest diversity was found in the natural soil ^(a)^, followed by dilutions 10^-1 (b)^, 10^-3^ ^(c)^, autoclaved soil ^(d)^ and dilution 10^-6^ ^(d)^ **(A)**. Fungal community α-diversity measurements based on ITS1 region from ITS. According to the HSD test (P<0.05) using Shannon index, all dilutions have statistical diferences in fungal community, except dilution 10^-6^ that had similar index to 10^-3^ and autoclaved soil, with a higher diversity in natural soil ^(a)^, folowed by dilutions 10^-1 (b)^, 10^-3 (c)^, 10^-6 (cd)^, and autoclaved soil ^(d)^ **(B)**. Control = non-inoculated plants, UD1022 = plants inoculated with wild-type B. subtilis, and UD1022^eps-TasA-^ = plants inoculated with mutant strain UD1022^eps-TasA-^. Letters in parenthesis indicate statistical differences, with “a” as the highest and “d” the lowest diversity index.

**Figure S4** Relative abundance of bacterial phyla. NS = natural soil; D1 = soil dilution 10^-1^, D3 = soil dilution 10^-3^, D6 = soil dilution 10^-6^, and AS = autoclaved soil. Control = non-inoculated plants, UD1022 = plants inoculated with wild-type strain, and UD1022^eps-TasA-^ = plants inoculated with mutant B. subtilis.

**Figure S5** Relative abundance of fungal phyla. NS = natural soil; D1 = soil dilution 10^-1^, D3 = soil dilution 10^-3^, D6 = soil dilution 10^-6^, and AS = autoclaved soil. Control = non-inoculated plants, UD1022 = plants inoculated with wild-type strain, and UD1022^eps-TasA-^ = plants inoculated with mutant B. subtilis.


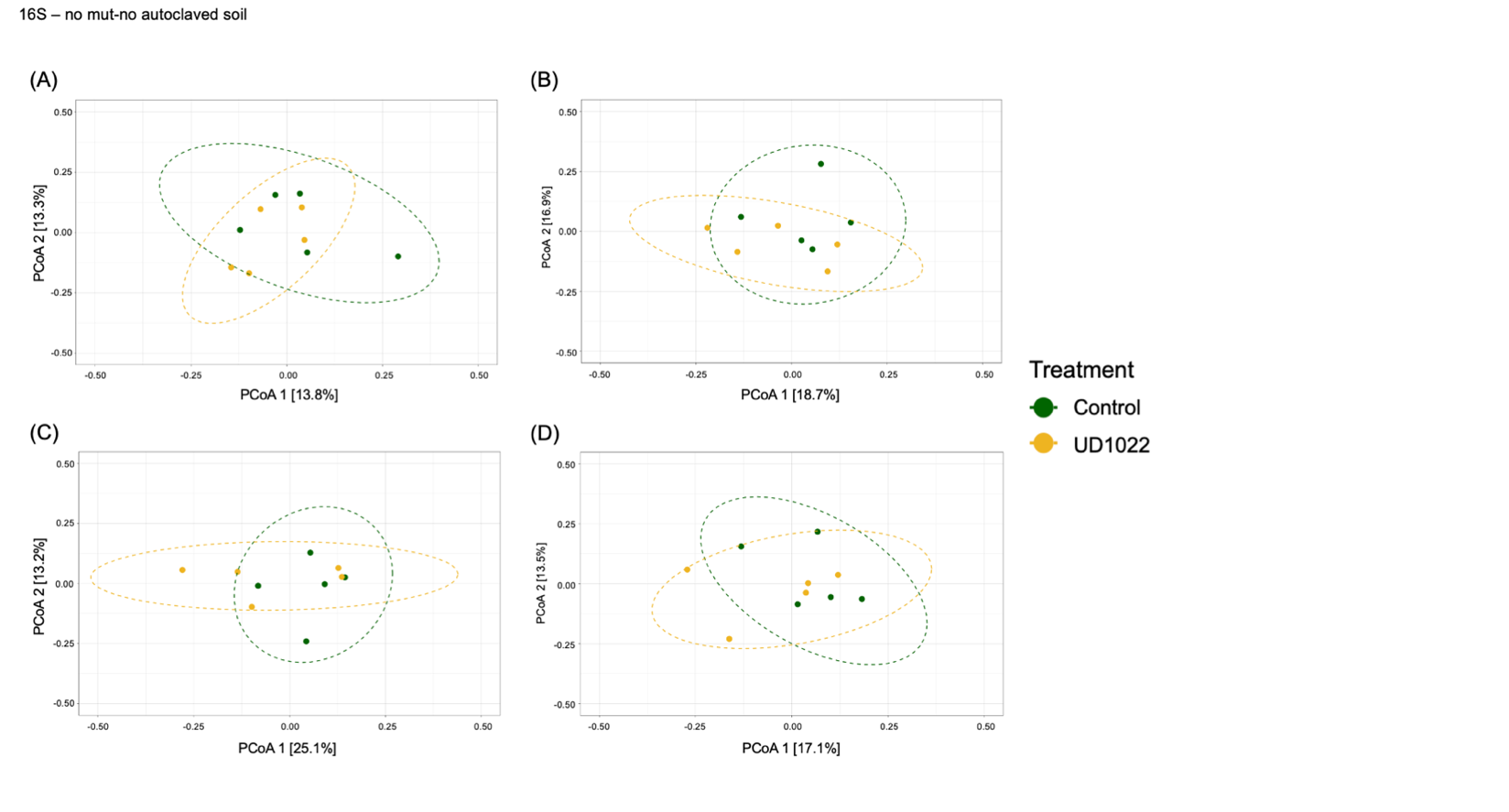


**Figure S6** Bacterial community β-diversity comparing the control with UD1022 treatment across the gradient of soil microbial diversity. PCoA method, Bray-Curtis’ distance, and statistical pairwise comparison by Adonis method (P<0.05, permutation=999) in natural soil; no significant differences (P= 0.111) **(A)**. **I**n soil dilution 10^-1^; no significant differences (P= 0.368) **(B)**. In soil dilution 10^-3^; no significant differences (P= 0.314) **(C)**. In soil dilution 10^-6^; no significant differences (P= 0.262) **(D)**. Control = non-inoculated plants and UD1022 = plants inoculated with wild-type B. subtilis.


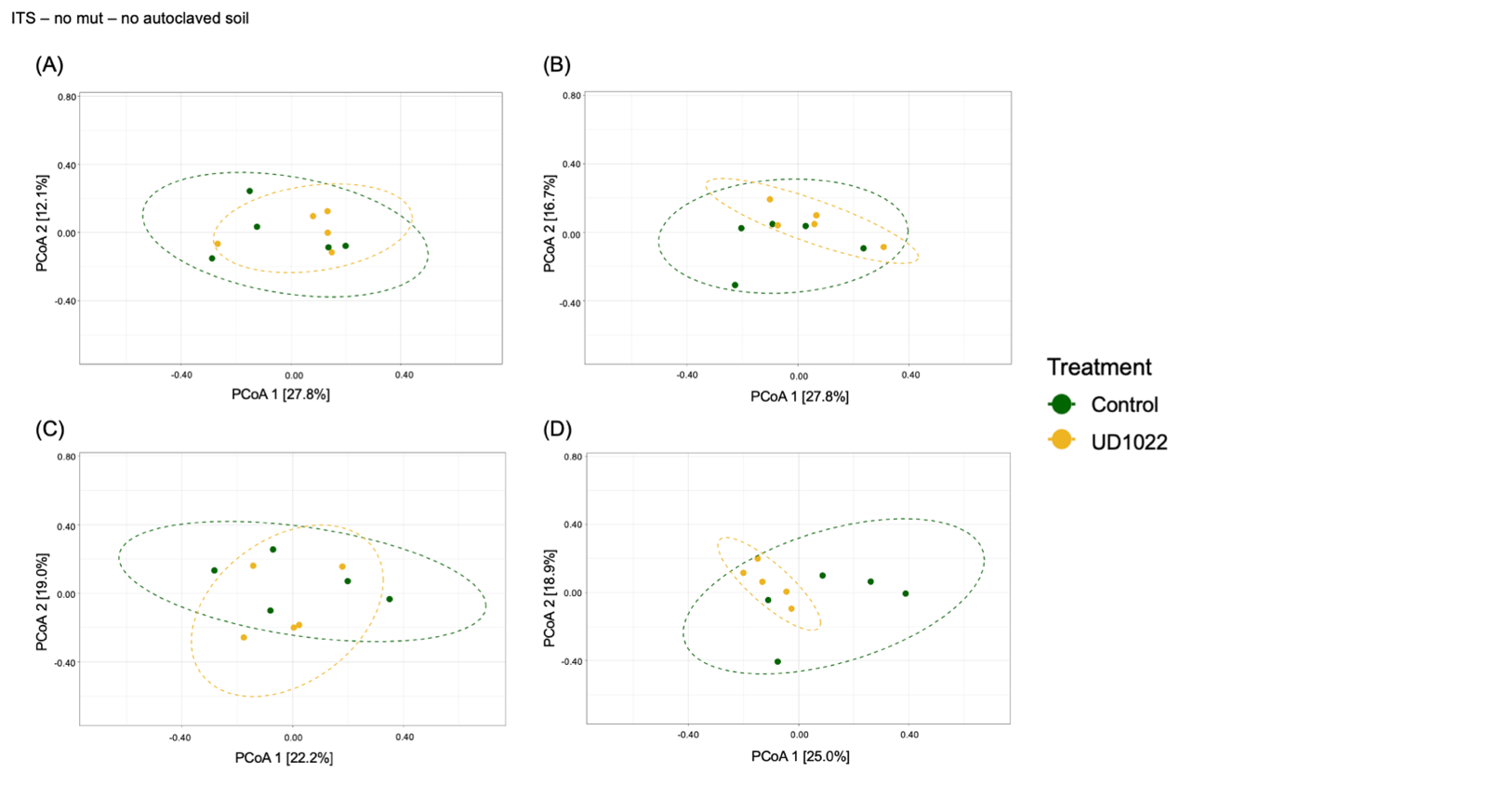


**Figure S7** Fungal community β-diversity comparing the control with UD1022 treatment across the gradient of soil microbial diversity. PCoA method, Bray-Curtis’ distance, and statistical pairwise comparison by Adonis method (P<0.05, permutation=999) in natural soil; no significant differences (P= 0.543) **(A)**. In soil dilution 10^-1^; no significant differences (P= 0.303) **(B)**. In soil dilution 10^-3^; no significant differences (P= 0.370) **(C)**. In soil dilution 10^-6^; no significant differences (P= 0.061) **(D)**. Control = non-inoculated plants and UD1022 = plants inoculated with wild-type B. subtilis.

**Figure S8** Relative abundance of bacterial and fungal genera comparing control with UD1022^eps-TasA-^ treatment. Bacterial relative abundance across soil dilutions **(A)**. Fungal relative abundance across soil dilution **(B)**. NS = natural soil; D1 = soil dilution 10^-1^, D3 = soil dilution 10^-3^, D6 = soil dilution 10^-6^, and AS = autoclaved soil. Control = non-inoculated plants, and UD1022^eps-TasA-^ = plants inoculated with mutant B. subtilis.


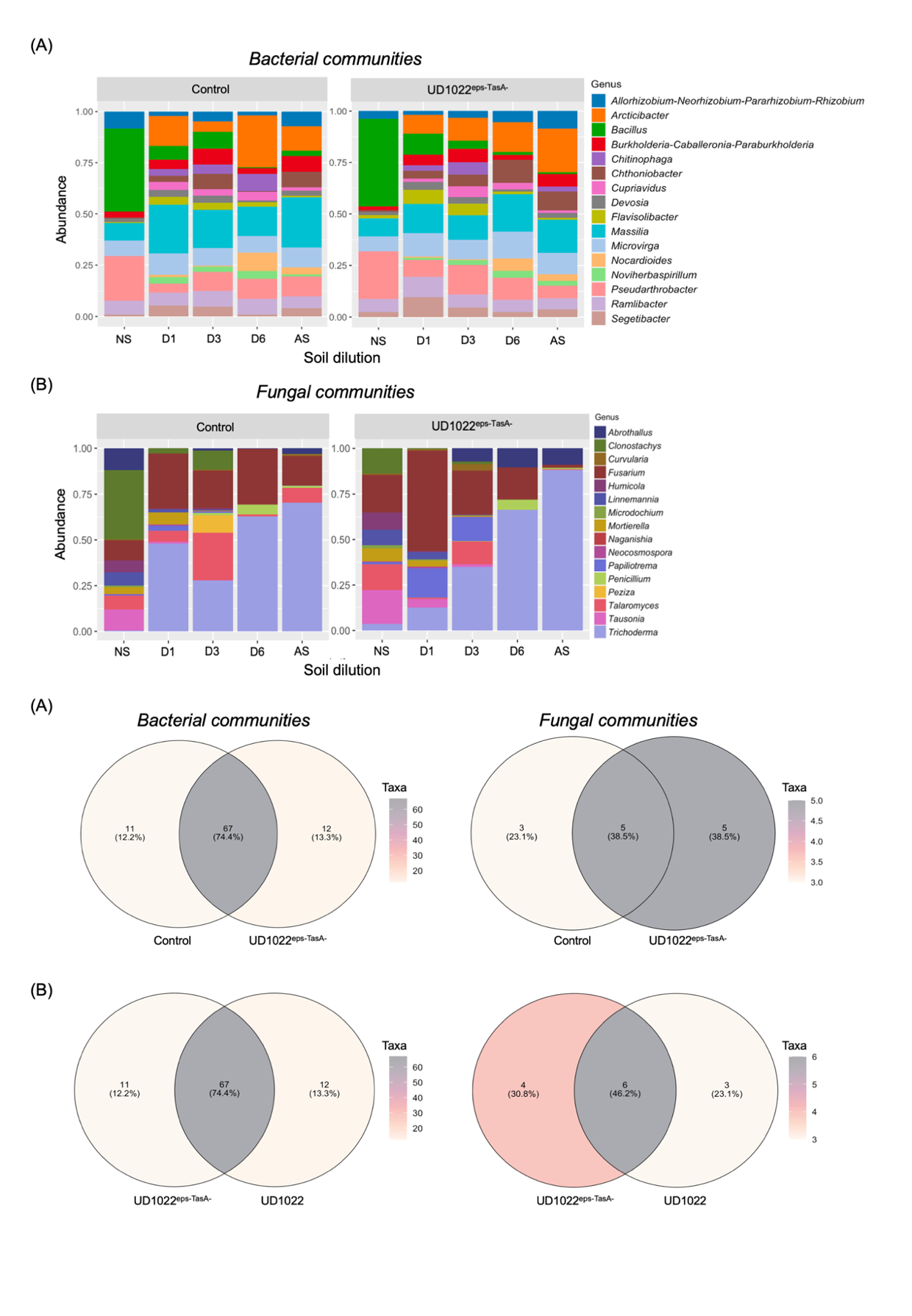


**Figure S9** Venn diagram of shared bacterial and fungi taxa between treatments. Shared bacterial and fungal taxa between control and UD1022^eps-TasA-^ treatment **(A)**. Shared bacterial and fungal taxa between UD1022^eps-TasA-^ and UD1022 treatment **(B)**. Control = non-inoculated plants, and UD1022^eps-TasA-^ = plants inoculated with mutant B. subtilis.


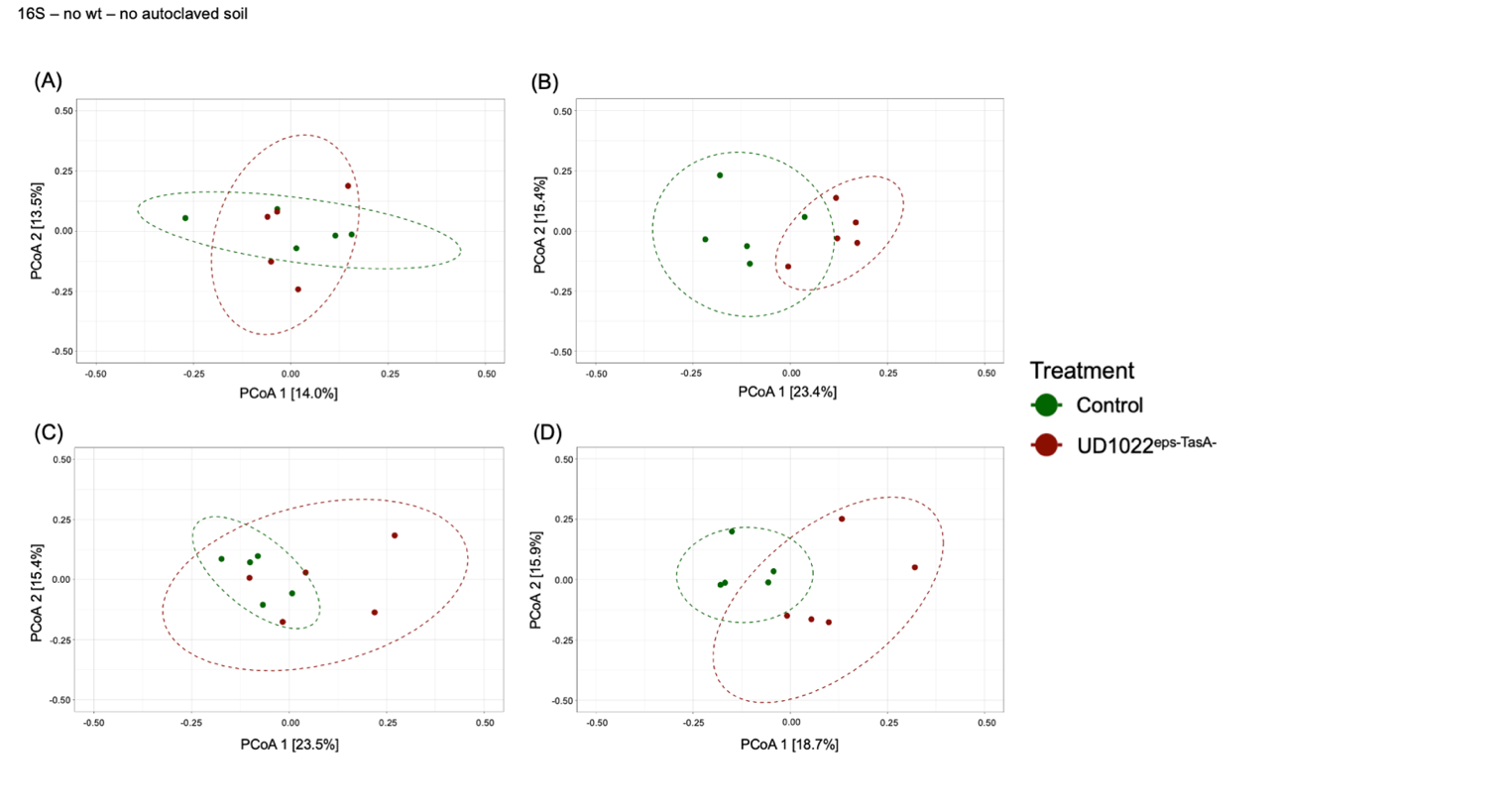


**Figure S10** Bacterial community β-diversity comparing the control with UD1022^eps-TasA-^ treatment across the gradient of soil microbial diversity. PCoA method, Bray-Curtis’ distance, and statistical pairwise comparison by Adonis method (P<0.05, permutation=999) in natural soil; no significant differences (P= 0.846) **(A)**. In soil dilution 10^-1^; no significant differences (P= 0.016) **(B)**. In soil dilution 10^-3^; no significant differences (P= 0.053) **(C)**. In soil dilution 10^-6^; no significant differences (P= 0.013) **(D)**. Control = non-inoculated plants and UD1022^eps-TasA-^ = plants inoculated with mutant B. subtilis.


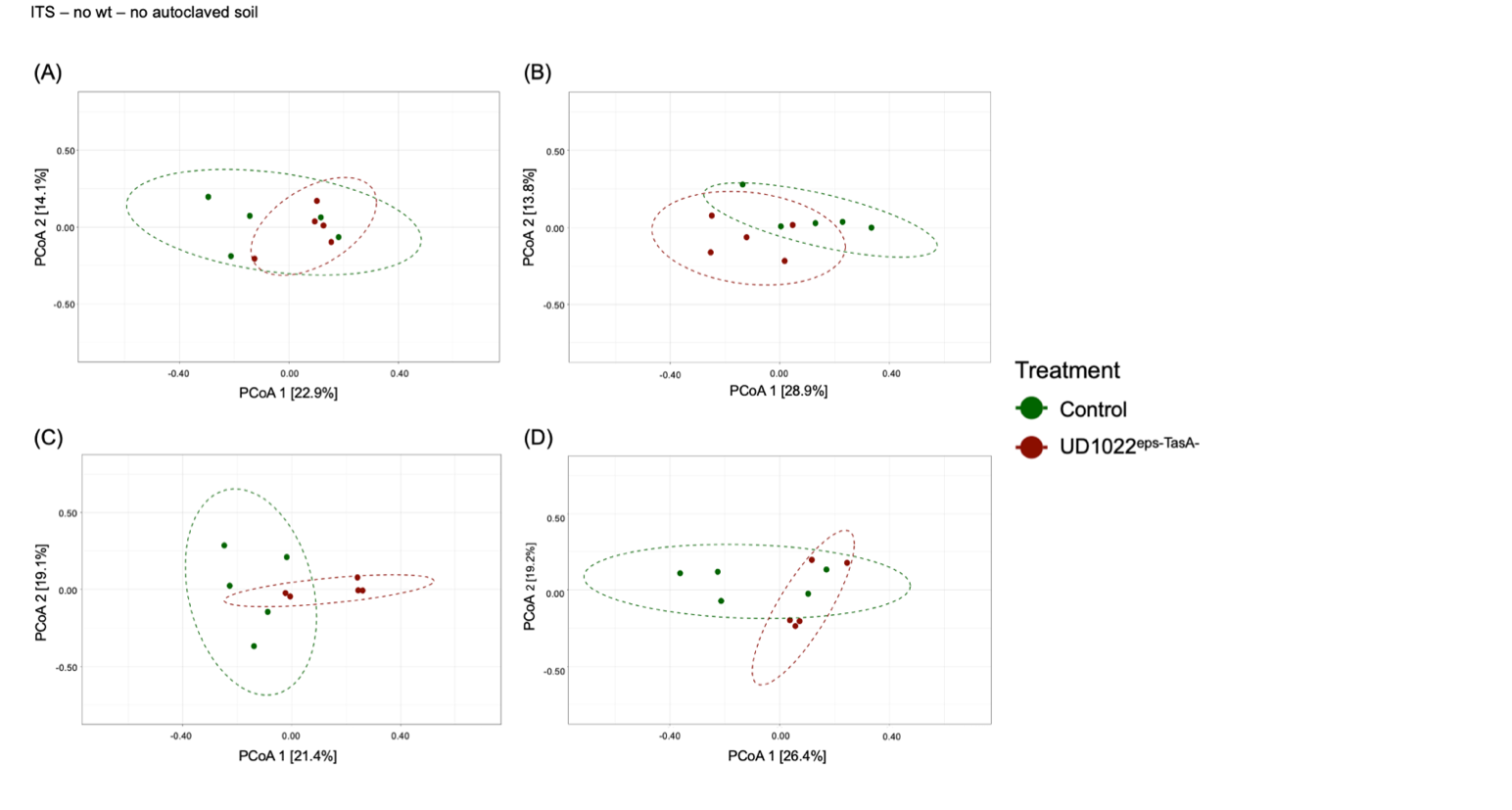


**Figure S11** Fungal community β-diversity comparing the control with UD1022^eps-TasA-^ treatment across the gradient of soil microbial diversity. PCoA method, Bray-Curtis’ distance, and statistical pairwise comparison by Adonis method (P<0.05, permutation=999) in natural soil; no significant differences (P= 0.330) **(A)**. In soil dilution 10^-1^; no significant differences (P= 0.057) **(B)**. In soil dilution 10^-3^; no significant differences (P= 0.023) **(C)**. In soil dilution 10^-6^; no significant differences (P= 0.113) **(D)**. Control = non-inoculated plants and UD1022^eps-TasA-^ = plants inoculated with mutant B. subtilis.

**
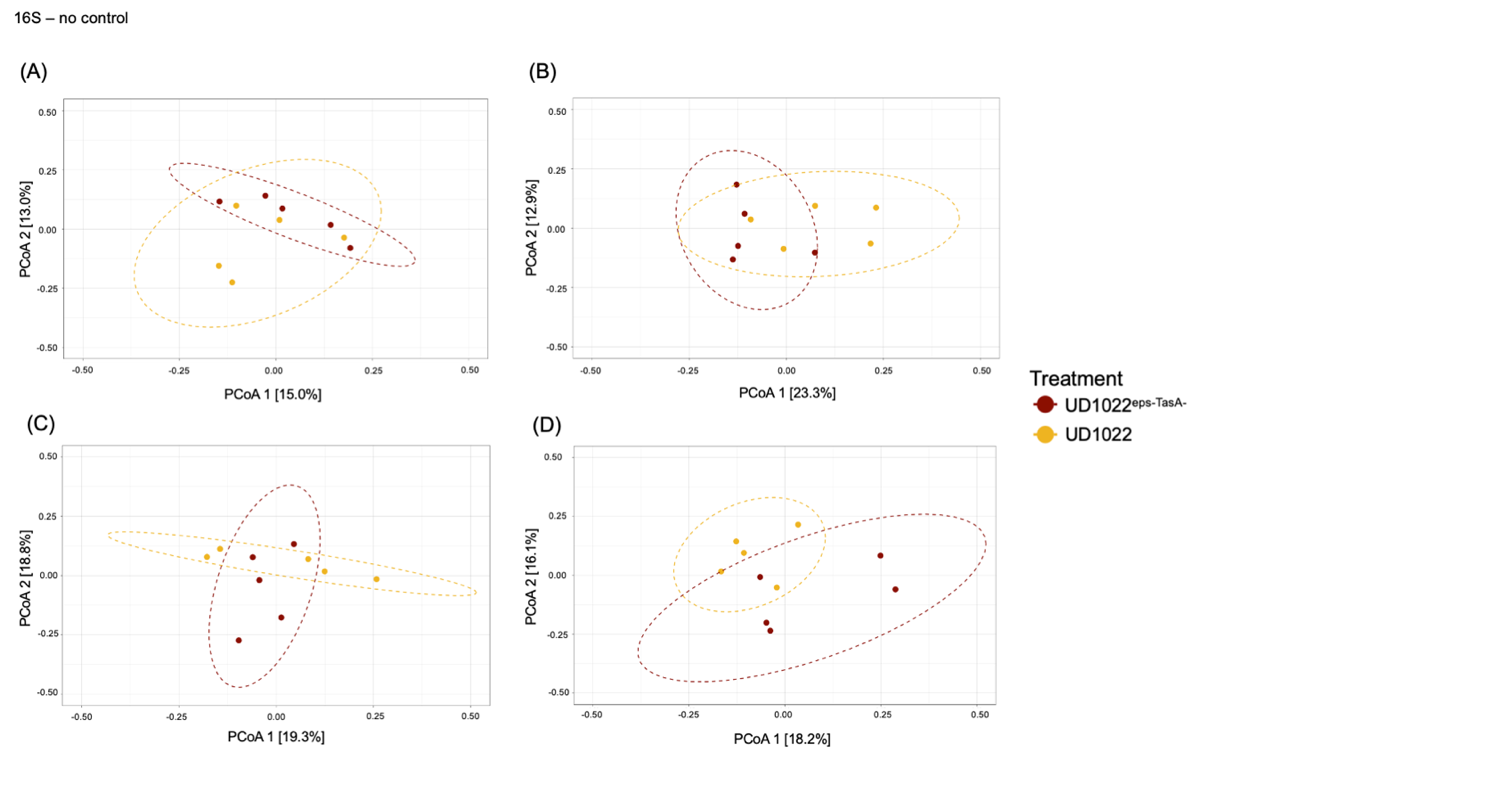
**

**Figure S12** Bacterial community β-diversity comparing the UD1022 with UD1022^eps-TasA-^ treatment across the gradient of soil microbial diversity. PCoA method, Bray-Curtis’ distance, and statistical pairwise comparison by Adonis method (P<0.05, permutation=999) in natural soil; no significant differences (P= 0.278) **(A)**. In soil dilution 10^-1^; no significant differences (P= 0.064) **(B)**. In soil dilution 10^-3^; no significant differences (P= 0.194) **(C)**. In soil dilution 10^-6^; no significant differences (P= 0.045) **(D)**. UD1022 = plants inoculated with wild-type B. subtilis and UD1022^eps-TasA-^ = plants inoculated with mutant B. subtilis.

**
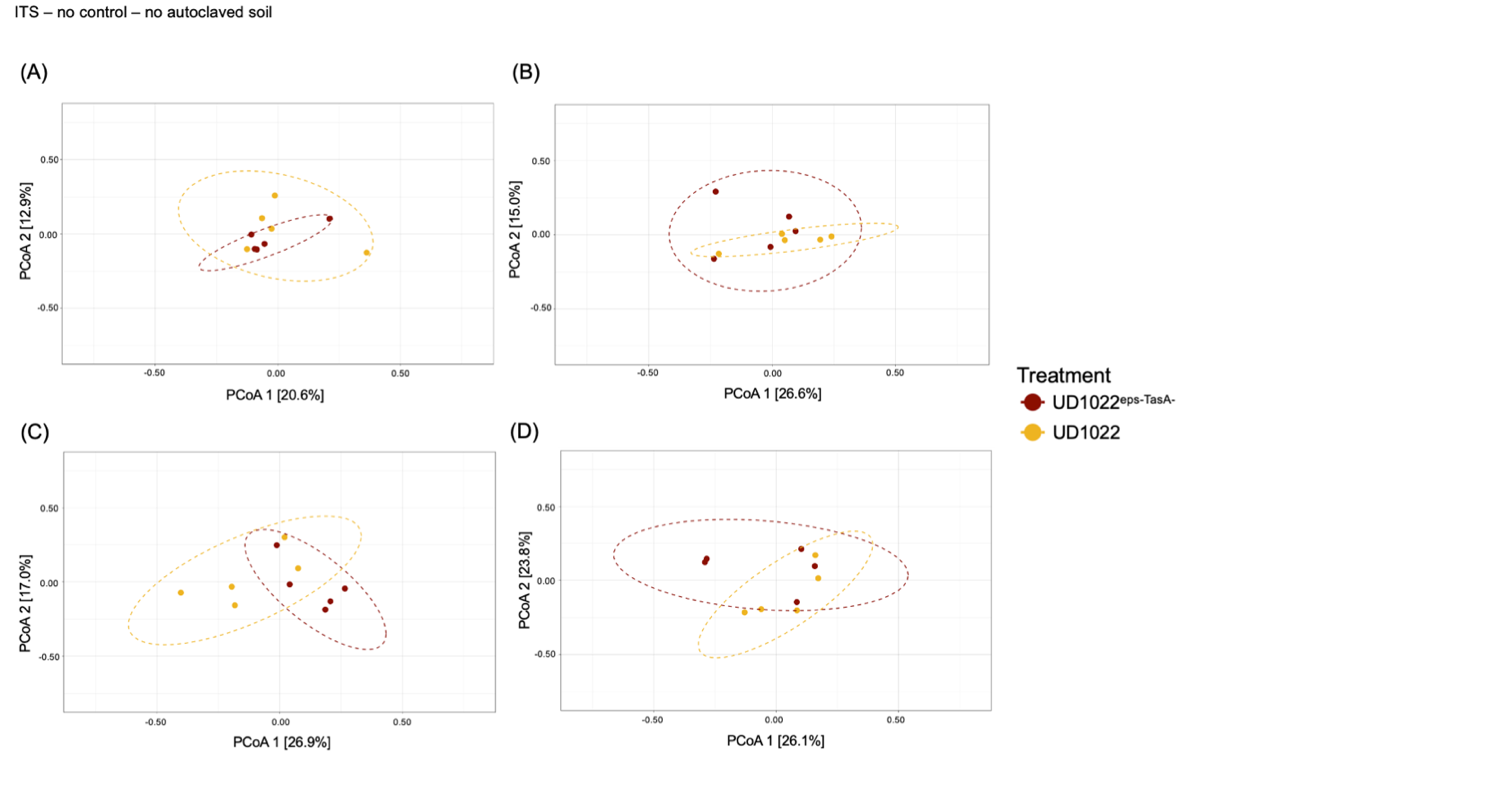
**

**Figure S13** Fungal community β-diversity comparing the UD1022 with UD1022^eps-TasA-^ treatment across the gradient of soil microbial diversity. PCoA method, Bray-Curtis’ distance, and statistical pairwise comparison by Adonis method (P<0.05, permutation=999) in natural soil; no significant differences (P= 0.701) **(A)**. In soil dilution 10^-1^; no significant differences (P= 0.119) **(B)**. In soil dilution 10^-3^; no significant differences (P= 0.070) **(C)**. In soil dilution 10^-6^; no significant differences (P= 0.168) **(D)**. UD1022 = plants inoculated with wild-type B. subtilis and UD1022^eps-TasA-^ = plants inoculated with mutant B. subtilis.

**Table S1** Co-occurrence network parameters for bacterial and fungal communities

| **Parameters** | **Control** | **UD1022^eps-TasA-^** | **UD1022** |
| --- | --- | --- | --- |
|  | *Bacterial Community network* | | |
| Number of nodes^a^ | 87 | 86 | 87 |
| Number of edges^b^ | 556 | 490 | 404 |
| Positive edges^c^ | 486 | 404 | 356 |
| Negative edges^d^ | 70 | 86 | 48 |
| Modularity^e^ | 0.43 | 0.46 | 0.52 |
| Number of communities^f^ | 4 | 4 | 5 |
| Network diameter^g^ | 7 | 6 | 6 |
| Average path length^h^ | 2.353 | 2.356 | 2.230 |
| Average degree^i^ | 6.39 | 5.70 | 4.64 |
| Average clustering coefficient^j^ | 0.27 | 0.24 | 0.25 |
|  | *Fungal community network* | | |
| Number of nodes^a^ | 33 | 36 | 33 |
| Number of edges^b^ | 196 | 273 | 229 |
| Positive edges^c^ | 164 | 232 | 201 |
| Negative edges^d^ | 32 | 41 | 28 |
| Modularity^e^ | 0.21 | 0.15 | 0.15 |
| Number of communities^f^ | 3 | 3 | 3 |
| Network diameter^g^ | 4 | 4 | 4 |
| Average path length^h^ | 1.657 | 1.558 | 1.662 |
| Average degree^i^ | 5.94 | 7.58 | 6,94 |
| Average clustering coefficient^j^ | 0.32 | 0.36 | 0.35 |

aMicrobial taxon (at Phylum level) with at least one significant (P < 0.001) and strong (Co-occurence > 0.001 or < -0.001) correlation;

bNumber of connections/correlations obtained by Co-occurence analysis;

cPositive correlation measured by PhyloSmith package v. 1.0.6 (Co-occurence function) (> 0.001 with P < 0.001);

dNegative correlation measured by PhyloSmith package v. 1.0.6 (Co-occurence function) (< -0.001 with P < 0.001);

eThe capability of the nodes to form highly connected communities, that is, a structure with high density of between nodes connections (inferred by Gephi);

fA community is defined as a group of nodes densely connected internally (Gephi);

gThe longest distance between nodes in the network, measured in number of edges (Gephi);

hAverage network distance between all pair of nodes or the average length off all edges in the network (Gephi);

iThe average number of connections per node in the network, that is, the node connectivity (Gephi);

jHow nodes are embedded in their neighborhood and the degree to which they tend to cluster together (Gephi).
